# Developing capacity in health informatics in a resource poor setting: lessons from Peru

**DOI:** 10.1186/1478-4491-7-80

**Published:** 2009-10-27

**Authors:** Ann Marie Kimball, Walter H Curioso, Yuzo Arima, Sherrilynne Fuller, Patricia J Garcia, Jose Segovia-Juarez, Jesus M Castagnetto, Fabiola Leon-Velarde, King K Holmes

**Affiliations:** 1 University of Washington, Seattle, Washington, 98195, USA; 2 Universidad Peruana Cayetano Heredia, Av Honorio Delgado 430 Urbanizacion Ingenieria-San Martin de Porres, Lima 31, Peru

## Abstract

The public sectors of developing countries require strengthened capacity in health informatics. In Peru, where formal university graduate degrees in biomedical and health informatics were lacking until recently, the AMAUTA Global Informatics Research and Training Program has provided research and training for health professionals in the region since 1999. The Fogarty International Center supports the program as a collaborative partnership between Universidad Peruana Cayetano Heredia in Peru and the University of Washington in the United States of America. The program aims to train core professionals in health informatics and to strengthen the health information resource capabilities and accessibility in Peru. The program has achieved considerable success in the development and institutionalization of informatics research and training programs in Peru. Projects supported by this program are leading to the development of sustainable training opportunities for informatics and eight of ten Peruvian fellows trained at the University of Washington are now developing informatics programs and an information infrastructure in Peru. In 2007, Universidad Peruana Cayetano Heredia started offering the first graduate diploma program in biomedical informatics in Peru.

## Introduction

Technology serves public health, and an important mission for public health schools today is to advance knowledge and provide training in 'appropriate technologies' for the control of diseases of poverty in developing countries [1]. Appropriate technologies must be developed, produced, delivered, and monitored within a comprehensive framework that takes into account the systems, the individuals, and the community [1].

In developed countries, biomedical and public health informatics have been taught over the last 20-25 years, with well documented successes and setbacks, providing good models for future course development and training [2]. Public health informatics can be defined as the systematic application of information and computer science and technology to public health practice, research, and learning [3].

In resource poor settings, informatics represents an important and emerging focus in healthcare settings, applicable, for example, to functional genomics, proteomics, clinical care, prevention, disease surveillance, and disease burden assessment, which are among the appropriate technological 'core competencies' for medical and public health research [1]. However, in developing countries, the need for training and retention of health professionals in informatics remains one of the greatest public health challenges. For instance, although a large proportion of genomic studies of microbial pathogens has involved pathogens of special importance in developing countries, involvement of researchers from developing countries in such projects has been quite limited, constraining timely development of bioinformatics capacity in developing countries [4,5]. Education in medical informatics skills in these regions is also inadequate, and levels of computer competence vary among medical students, doctors, nurses, and many other health care professionals [6]. In 2003, Samuel et al. reported that only 52% of medical students in Tanzania felt that they understood the basic terminology and concepts of computing [7]. In a 2002 study conducted by Horna et al., 40% of a sample of medical students in Peru reported lack of proficiency in the use of the internet [8]. With the growing use of the internet to gain access to vital health information and for research [9], informatics training and computer competency are essential for all health professionals.

Latin America, like other areas of the developing world, suffers from high incidence of HIV/AIDS, tuberculosis, and malaria, and of other important but neglected infectious diseases [1]. Improving information systems and overcoming the digital divide (internet connectivity and computing capacity) will support the effort to reduce the morbidity and mortality associated with these diseases [1,10]. For example, informatics systems such as the Perinatal Information System (Sistema de Información Perinatal [SIP]), developed by the Latin American Center for Perinatology and Human Development (CLAP-PAHO/WHO) [11], have shown that such systems are useful for quality assurance of care and monitoring of health indicators [12]. More recently, training programs have also been implemented in Latin America; for instance, the American Medical Informatics Association's (AMIA) 10 × 10 program has been translated and adapted into Spanish by Hospital Italiano at Buenos Aires [13].

In this paper, we present a case study from Peru that utilizes a comprehensive framework and provides training in appropriate health informatics technologies. The objective of this paper is to provide a successful and sustainable model of a health informatics training program based upon a comprehensive framework and use of technologies to support medicine and public health in Peru. The lessons learned are intended to assist other developing countries, especially those in Latin America, that wish to enhance their informatics capabilities.

## Development of the AMAUTA Global Informatics Research and Training program in Peru

Peru is a developing Andean country on the west coast of Latin America. Its territory embraces a diverse population of 27 900 000 with annual per capita gross domestic product (GDP) of US$ 8400 [14]. Only 4.3% of the GDP is devoted to Peru's health care sector [15]. While considered a middle-income country, Peru remains excluded by the 'digital divide', with only 9.7% of its citizens owning private telephones, and only 5.1% having a computer with internet connection [16]. However, the proliferation of public 'cabinas' (internet cafés) has provided some alternative internet access, and has become a research topic of its own for public health informatics [17]. In addition, the revolution of cell phones has also come to facilitate data collection and evaluation in public health [18].

The AMAUTA Global Training in Health Informatics program (AMAUTA is a Quechua word meaning a person of great wisdom, one who knows and who teaches) was developed in 1999 to train Peruvian health care professionals and students in the use, development and application of informatics. This collaborative program is an institutional partnership that has involved Universidad Peruana Cayetano Heredia (UPCH) and University of San Marcos of Peru and the University of Washington (UW), Seattle, Washington USA, with the support of the Fogarty International Center (FIC), U.S. National Institutes of Health (NIH). University of San Marcos was partner in the first five years of the Research and Training program and remains a collaborator rather than a partner since 2005.

UPCH is recognized as the Peruvian university with the highest levels in research and training in medicine and public health. UPCH has been building capacity in research, training, infrastructure and human resources while developing key partnerships, with a long history of funding support from the FIC and other NIH-funded projects, as well as the Wellcome Trust, the World Health Organization (WHO), the European Community, and other regional and national funds (e.g. the Peruvian Science and Technology Program). In recent years, UPCH has been making efforts to develop a structure for online education, targeting students and professionals with difficulties in accessing the classic educational system. Thus, the current goals of the AMAUTA program include training of core professionals in public health/medical informatics and strengthening the library and health information resource capabilities and accessibility at UPCH. The program aims to comprehensively strengthen the informatics training and capacity framework of individuals, systems, and the community.

The ongoing AMAUTA Program collaboration has been 'rolled out' in a step wise fashion, from individuals to institutions including: 1) short course offerings in Peru to identify needs and collaborators and potential trainees; 2) longer term graduate level training in informatics for core UPCH faculty; 3) digitization of the UPCH library; and 4) targeted short term training at the UW for critical need areas such as library science (in partnership with the UW Health Sciences Libraries) and laboratory informatics. The collaborative program has made available a range of informatics applications (i.e. public health, medical, and bio-informatics) and offers training opportunities in electronic data collection methods; resource access at UPCH; library and information science and appropriate resource development; open-source learning management systems (e.g. Moodle); geographic information systems for monitoring disease incidence and outbreaks; and bioinformatics training related to the genomics of infectious diseases (e.g. HIV, syphilis, tuberculosis, and malaria).

AMAUTA has from the start focused on long term capacity building, training individuals to strengthen the informatics workforce and building capacity at the institutional system level. Individual training has included both short and long term training. Short term courses offered in Peru involve: 1) intensive two week introductory courses on public health informatics and medical informatics and; 2) training of trainers, i.e. training of UPCH faculty members to teach an introductory informatics course to their own students. Long term training is offered at the UW, where key UPCH faculty members are trained as researchers and instructors of informatics through certificate, masters, post-masters, doctoral, and post-doctoral training programs, to enhance the core informatics capacity at UPCH and Peru. This flexible range of opportunities allows both busy professionals and young students to receive informatics training that suits their needs and schedules. The training of competent native scientists is essential, because it allows research communities to grow and conduct research that contributes to local improvements in public health and medicine [9].

So far, four short courses have been organized in Lima in 2000, 2001, 2005, and 2008, offering training in informatics to more than 200 graduate level students. The first two courses covered topics in medical informatics and public health informatics through 60 in-class hours of lecture, group exercises, and field/laboratory training [19]. The last two courses covered the same content over 48 hours of coursework each [5]. Participants performed considerably better on the informatics knowledge test after the course, and overall acceptance of the course was ranked as very good to excellent, while the usefulness of the course was rated as very good [5]. Follow-up evaluation six months after the course indicated no decline in evaluation scores [5]; additional evidence of the AMAUTA training program's positive outcomes has been described elsewhere [19]. Furthermore, there are prospects for continuing growth; for example, UPCH offered the first Graduate Diploma Program in Biomedical Informatics in Peru in 2007, led by former AMAUTA trainees [20].

At an institutional systems level, the AMAUTA program has been designed to be sustainable and to encourage the retention of native scientists in Peru. Visiting scholars engaged in long term training at the UW are required to return to Peru, as explained by the training program's offer letter; in addition, both the trainees' visa and financial support at UW expire at the time of training completion. The program generally guarantees a position at UPCH or another Peruvian institution upon completion, and this career advancement opportunity has helped prevent the migration of the skilled workforce from Peru (i.e. 'brain drain'). Indeed, eight of ten long-term trainees have returned to Peru and many continue to be engaged in the public sector. Institutionalization of capacity is also a key component of sustainable capacity building. Because individuals with informatics skills are highly prized by industry and by non-governmental organizations (NGOs) in Peru, negotiating realistic salaries and careers in university settings proved to be a pivotal area of endeavor for the AMAUTA program.

Since 2000, the program has been successful in identifying internal and external resources and identifying ongoing funding from complementary sources (e.g., Asia Pacific Economic Cooperation [APEC]) for scholar support; UPCH was also the only successful foreign submission for a Fogarty 'Framework Programs for Global Health' research and training grant in 2005 [21]; and a returning scholar recently successfully competed for an R01 Global Research Initiative Program (GRIP) grant [18].

At UPCH, AMAUTA has forged ongoing and novel partnerships between trainees and librarians to develop sustainable databases and interfaces for Peruvian institutions. For instance, library/resource enrichment (e.g. state-of-the-art computers with wireless internet connectivity) has recently been completed at UPCH with support from former trainees. In addition, AMAUTA has taken innovative advantage of inexpensive and abundant 'cabinas' and cellular telephones throughout much of Peru. Real-world use of these existing technologies allows for practical training appropriate for community-level public health [17]. Reflecting the institutional partnership, instructors and trainees at UW and UPCH communicate via teleconference and videoconference on a regular basis for updates on the progress of trainees and sharing of future plans.

Lastly, the institutionalization of informatics capacity at UPCH has enhanced the biomedical research capacity at UPCH through improved information access and infrastructure. AMAUTA encourages interdisciplinary collaborations between computer scientists and biomedical scientists, allowing integration of informatics training with ongoing public health and biomedical research programs. Continuation of the program has been possible through shared program management and well-functioning technical oversight and coordination. The close professional relationships of Peruvian faculty, librarians and students with their UW counterparts has enriched the program and resulted in new research and teaching collaborations at both institutions in ways not envisioned at the beginning of the program.

## Discussion

This brief overview of the AMAUTA Global Informatics Research and Training Program presents an example of a successful health informatics training program in a developing country. AMAUTA has retained a high proportion of Peruvian scientists in the discipline of informatics in Peru. Our experience agrees with recent findings that capacity building is an important strategy in improving health systems in developing countries [1,11]. In addition, we have learned that a combination of short and long term strategies directed at both individual and institutional capacity have helped to develop the local health research enterprise [9]. AMAUTA's flexibility and ability to understand the local context have been major strengths of the program.

In our experience, a stable funding base over a long period of time (optimally at least for ten years) has been critical for the development, nurturing and maintenance of this collaborative program. The fact that former AMAUTA trainees continue to compete successfully as principal investigators for independent funding from several sources of funding, both nationally and internationally (e.g. from the UW and the U.S. NIH), provides evidence of the sustainability and rigor of the training program, and of the building of local capacity. And, once a stable funding base and infrastructure have been established, it is important to pass on the leadership; UPCH, and not the USA-based UW, has now taken the lead to direct the project.

Growing interest in global health should increase the opportunity for such collaborative partnerships aimed at improving medicine and public health. Public health schools elsewhere could jump-start their efforts to conduct informatics and other appropriate technology training for developing countries by partnering with other foundations and institutions [1]. While AMAUTA has been supported through FIC, another possible source of support could include the Pan American Health Organization (PAHO; ), one of the most effective public health agencies in the world, which could assess, enable, and evaluate application of appropriate informatics technologies in Latin America. In addition, in the past few years, the private sector and other non-public entities have made unprecedented commitments to solving neglected public health problems, and can foster development of informatics training. The Bill & Melinda Gates Foundation  and Rockefeller Foundation  are just a few notable examples. The former has funded AMIA to develop a global biomedical and health informatics fellowship program  while the latter has focused on informatics training through disease surveillance networks .

Continuous support from the university authorities has been essential for the success of the AMAUTA program. While attracting qualified native professionals trained abroad is a continuous challenge, the program's ability to retain 80% of its trainees in informatics in Peru has been associated with careful selection of candidates, maintaining the connection and collaboration between partner institutions, and fostering an enabling environment [22]. The program's strength was enhanced by the decision of the FIC to permit in-country research support for each returning scholar, and the collaboration with UW enhanced the ability of UPCH to strengthen its own portfolio of research projects leading to additional support for institutionalization.

## Conclusion

Collaborative partnerships between countries can optimize training opportunities and human and technological resources; such partnerships will undoubtedly become more important as globalization continues. In this context, we now require a more integrated, practical approach that includes acquisition of 'real-world' skills in various technologies [1]; the use of cell phones and 'cabinas' are just a few examples that we found available and appropriate for Peru [23]. By supporting both short and long term training programs focused on capacity building of individuals and institutions, sustainable training programs for informatics and other appropriate technologies can be formed in developing countries.

## Competing interests

The authors declare that they have no competing interests.

## Authors' contributions

AMK conceived of the AMAUTA Program, the scope and substance of the article and revised and edited the manuscript and coordinated finalization among authors. WHC participated in the design and coordination of the program, helped draft the manuscript, and revised it critically for important intellectual content. YA drafted and edited the manuscript and revised it critically for important intellectual content. SF helped to design and coordinate the AMAUTA Program and reviewed the manuscript critically. PJG, JSJ, JMC and FLV helped draft the manuscript and revised it critically for important intellectual content. KKH helped to design and coordinate the development of the AMAUTA Program, and reviewed and edited the manuscript. All authors read and approved the final manuscript.
